# An immune reaction may be necessary for cancer development

**DOI:** 10.1186/1742-4682-3-6

**Published:** 2006-02-03

**Authors:** Richmond T Prehn

**Affiliations:** 1 Department of Pathology University of Washington Seattle, WA, USA

## Abstract

**Background:**

The hypothesis of immunosurveillance suggests that new neoplasms arise very frequently, but most are destroyed almost at their inception by an immune response. Its correctness has been debated for many years.

In its support, it has been shown that the incidences of many tumor types, though apparently not all, tend to be increased in immunodeficient animals or humans, but this observation does not end the debate.

**Alternative model:**

There is an alternative to the surveillance hypothesis; numerous studies have shown that the effect of an immune reaction on a tumor is biphasic. For each tumor, there is some quantitatively low level of immune reaction that, *relative to no reaction*, is facilitating, perhaps even necessary for the tumor's growth *in vivo*. The optimum level of this facilitating reaction may often be less than the level of immunity that the tumor might engender in a normal subject.

**Conclusion:**

The failure of a tumor to grow as well in the normal as it does in the immunosuppressed host is probably not caused by a lack of tumor-cell killing in the suppressed host. Instead, the higher level of immune response in a normal animal, even if it does not rise to tumor-inhibitory levels, probably gives less positive *support *to tumor growth. This seems more than a semantic distinction.

## Introduction

It is now almost 50 years since the first convincing demonstration that implantation of most MCA (3-methylcholanthrene)-induced mouse sarcomas into animals of the same inbred strain as the animal of origin could induce a tumor-specific, growth-inhibiting immunity [1]. The phenomenon proved general; tumors that were induced by other known oncogens, such as other chemical carcinogens, radiation or oncogenic viruses, were usually demonstrably immunogenic in transplantation tests. It was also observed that, at least in the case of MCA-in-paraffin-induced tumors, the degree of immunogenicity tended to be directly related to the concentration of the inducer [2-4], suggesting that sporadic, spontaneous tumors might characteristically have little or perhaps no immunogenicity. This point will be further discussed.

When immunogenic MCA -induced tumors were passaged by transplantation through syngeneic hosts, the immunogenicity proved to be surprisingly stable from one tumor generation to the next [5]. Although both Bartlett [6] and Bubenik [7] demonstrated some selective effects related to immunogenicity, highly immunogenic tumors usually remained highly immunogenic and those of lesser immunogenicity tended to remain as such. However, sometimes a tumor appeared to either gain or lose an aspect of immunogenicity; this could be either a change in immunizing ability [8] or a change in susceptibility to the effect of immunity on the tumor's growth [5]; gain or loss in either of these parameters was often compensated by an opposite change in the other [5]. Although the tumors could change their immunogenic characteristics with time, their surprising overall stability suggested that the changelings had little selective advantage [5]. The expectation that passage would select for nonimmunogenic tumor variants, predicted by the immunosurveillance hypothesis, was at best only partially realized.

**Figure 1:**
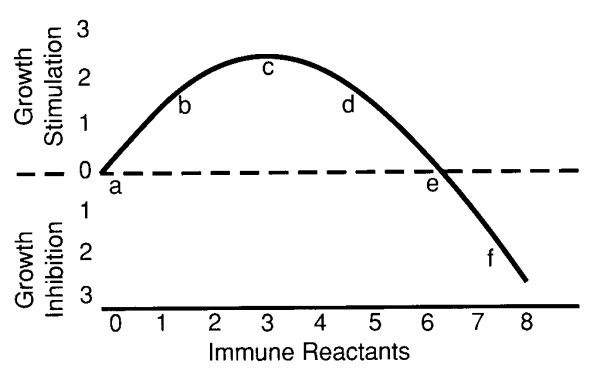
Relationship between tumor stimulation or inhibition and the relative quantity of the immune reactants.

Almost a quarter of a century ago, a wide range of observations, including a possible benefit to the fetus of an anti-fetal immune reaction, suggested that immunity might sometimes serve to stimulate or facilitate rather than inhibit tumor growth [9,10]. This hypothesis was soon supported by experiment. It was shown that a syngeneic, immunogenic tumor-implant in a thymectomized and irradiated mouse was stimulated to grow by mixing the inoculum with a small proportion of specifically immune, as compared with nonimmune, spleen cells. However, larger proportions of the same immune-cell population inhibited tumor growth [11]. Owing to the radiation and thymectomy, this finding was believed to be unaffected by the host's native immune mechanisms. Thus, it was concluded that the immune response affected the growth of syngeneic tumor implants in mice biphasically; a quantitatively small immune reaction would facilitate tumor growth, but a larger reaction would be inhibitory. An apparently analogous phenomenon was seen *in vitro *when tumor cells were exposed to varying numbers of specifically immune lymphoctes [12]. This putative relationship, as illustrated in a previous publication [13], is shown in Fig. 1.

Presumably an immune reaction must be of small magnitude before it becomes large. This presumption is supported by the observation that 5 days after implantation of an immunogenic, MCA-induced tumor, peripheral blood lymphocytes were stimulatory to tumor growth in vitro, but by 12 days they had become inhibitory [14]. This result seems to challenge the surveillance hypothesis: could there be surveillance of incipient tumors if incipient immune reactions are weak and therefore in the tumor-facilitating range? So does the surprising stability of tumor immunogenicity on passage through numerous transplant generations [5], discussed above.

### The biphasic effect of immunity on tumor-induction by applied oncogens

The biphasic effect of the immune reaction on syngeneic tumor-implants in mice does not necessarily indicate the effect of immunity on primary, untransplanted tumors of the type seen in the clinic, *i.e*. on untransplanted tumors growing in their autochthonous hosts. It was soon apparent that the mouse in which a tumor originated was immunologically very different from an animal that received a tumor as an implanted fragment. The original tumor-bearer was not noticeably immunized by the primary *in situ *tumor and could only be immunized by repeated subsequent implantations of that same tumor [15]. This was true even if the *in situ *tumor were subsequently shown, *when transplanted to a naive animal*, to be highly immunogenic. Furthermore, a subsequent inoculation of the same immunogenic tumor usually grew *better *in the primary mouse than it did in mice that had not previously been exposed to the tumor but had received a comparable amount of carcinogen [16,17]. The mechanistic basis for the failure of the primary tumor-bearer to develop tumor-inhibiting immunity remains uncertain, but may well be caused by a partial T-cell tolerance [18] and/or a weak tumor-facilitating immune reaction induced by the manner of initial antigen-presentation. Irrespective of the mechanism, the essential point is that the primary tumor-bearer does not seem to develop a tumor-inhibiting immune reaction, which would seem to be necessary for surveillance. However, the question remains: does the immune reaction actually facilitate the growth of an *in situ *tumor in the autochthonous host? Many experiments, albeit most from my own laboratory, give strong evidence that the answer is yes.

The first experiment I have selected for discussion examined the effect of immunity on the behavior of primary, untransplanted, *in situ *tumors in normally immunocompetent mice. Prehn and Bartlett [19] showed that when sarcomas were induced subcutaneously by surgically-implanted paraffin wafers impregnated with a uniform concentration of MCA (3-methylcholanthrene), the 1 54 resulting sarcomas possessed a wide range of immunogenicity levels as judged by the growth of implants of these tumors in specifically immunized mice. Curiously, these immunogenicity levels fell into two distinct clusters, one of higher average immunogenicity and one of lower, with relatively few intermediates. In the primary animals, undisturbed sarcomas that were shown in subsequent transplantation studies to be of intermediate immunogenicity grew significantly faster (had shorter latencies) than tumors belonging to either the greater or the lesser immunogenicity cluster. Apparently, *immune selection favored an intermediate level of immunogenicity*. However, the average latency of primary sarcomas in the more highly immunogenic cluster was significantly *less *than the average latency of those in the lower. In other words, the highly immunogenic tumors tended, on average, to grow significantly faster than the less immunogenic ones when undisturbed in the original immunocompetent host, *but not as fast as those of intermediate immunogenicity *[19].

As part of the same experiment, tumors originally induced in immunologically-isolated intraperitoneal diffusion-chambers also exhibited the same two immunogenicity clusters, though the tumors in the higher cluster were significantly more immunogenic than those in the higher cluster from the subcutaneous induction study [19]. This observation reinforced the interpretation of the previous findings: among the tumors that were induced subcutaneously, the immune response *had indeed reduced *the higher tumor-immunogenicities toward an optimum level for growth. The optimum immunogenicity for facilitating subcutaneous tumor growth was apparently intermediate between the high and the low clusters.

These facts are consistent with the interpretation that, at least in the system examined, a modest immune reaction against primary undisturbed MCA-induced sarcomas stimulated or facilitated the tumor's growth. A modest level of immunogenicity was associated with the tumors that had the shortest latencies; the tumors of least immunogenicity had the longest latencies. These data do not fit easily with the immunosurveillance hypothesis. "Immunoediting" seemingly took place [20], but it apparently resulted in tumors that grew best in the presence of the intermediate rather than the lowest level of immune response. According to the immunosurveillance hypothesis, the tumors of least immunogenicity would have been expected to exhibit the shortest latencies [21].

A second experiment approached the same problem by directly varying the immune capacities of the hosts rather than by assessing the immunogenicities of the tumors. This was done by restoring to varying extents the immune capacities of mice that had been exposed to radiation and thymectomy. The restoration was accomplished by injecting different numbers of normal adult spleen cells intraperitoneally prior to the standard exposure to subcutaneously placed MCA. Moderate restoration of the suppressed immune capacity resulted in more tumors at a given time-point than did either maximal *or minimal *restoration. Note that the moderate restoration, in all probability, provided an immune capacity less than that to be found in a normal, fully immunocompetent animal [22].

Another experiment consistent with a biphasic immune effect on the development of MCA-induced tumors differed in that one of the experimental variables was the carcinogen concentration. I have already cited work indicating that the average immunogenicity of MCA-induced tumors tends to be directly related to the MCA concentration in the paraffin wafers. Marked differences in susceptibility to MCA-induced sarcogenesis had also been observed among various inbred strains of mice. L. Prehn and E. Lawler took advantage of these observations to show that the mouse strain most susceptible to oncogenesis with a high concentration of MCA was least susceptible with a low one, *and vice versa *[23]! Furthermore, with either concentration of MCA, the most susceptible mouse strain was made *more resistant *to tumor induction by immunosuppressive radiation, but the least susceptible strain was made more susceptible [24]. Both these experiments again suggest that the optimal immune response for facilitating the growth of *in situ *autochthonous, MCA-induced tumors was intermediate in magnitude between the highest and the lowest; it was certainly not the lowest, as the surveillance hypothesis predicts. (It must be noted that these results were not confirmed by Bernfeld and Homburger, probably owing to their use of MCA in liquid oil rather than as a solid wafer in paraffin [25]. Stutman found no obvious relationship between the dosage of MCA when administered in oil and the magnitude of the resulting tumor's immunogenicity [26]).

Mouse mammary tumors induced by the mouse mammary tumor virus show little or no immunizing ability when transplanted into mice carrying that virus. However, mammary tumors induced by MCA are highly immunogenic. Martinez [27] showed that newborn thymectomy *lowered *the incidence of virus-induced mammary tumors, but Johnson [28] reported that early thymectomy *accelerated *the appearance of chemically induced ones. Although from different laboratories, these combined results again suggest that immunosuppression, in this case by newborn thymectomy, favors the growth of more highly immunogenic *in situ *tumors while *inhibiting the development of tumors of lesser immunogenicity*. Again, the optimal immune capacity for tumor growth was apparently greater than zero.

Thymectomy at 3 days of age, in contrast to thymectomy either at birth or at 7 days, causes hyperplastic autoimmune lesions and increases susceptibility to subsequent chemical carcinogenesis [29]. This increase in susceptibility occurred only with low concentrations of MCA; with higher concentrations, the 3-day thymectomy was inhibitory. Again, the data suggest that an increased immune capacity, as evidenced in this case by the autoimmune diseases, facilitated growth of only the weakly immunogenic tumors produced by a low concentration of chemical; the highly immunogenic tumors, produced by higher concentrations of MCA, were relatively inhibited.

Outzen altered the immune capacities of mice by giving varied dosages of irradiation [30]. He then transplanted on to them syngeneic skin that had been exposed to a moderate dosage of MCA. Papillomas appeared earlier and most frequently in the skin grafts on those animals that had been exposed to an *intermediate *dosage of radiation. This experiment had the advantage that the host animals were not directly exposed to any possible immunosuppressive effects of MCA, nor was the skin exposed to radiation. Again, in this experiment, oncogenesis was best facilitated in animals that had a diminished but still positive immune capacity.

Ryan *et al*. [31] showed that antibodies to skin could be induced in mice by injecting skin; syngeneic skin produced low levels, but xenogeneic produced very high ones. It was then shown that syngeneic injections, which produced low levels of antibody, *promoted *the appearance of papillomas in carcinogen treated skin, but xenogeneic injections failed to do so.

Viral oncogenesis also seems to be subject to a biphasic effect of immunity. Murasko and Prehn [32] varied the immunizing dosage of inactivated Moloney murine leukemia virus and studied the effect on the induction of tumors by subsequent inoculation with a standard dosage of active virus. Mice immunized with high dosages developed significantly fewer tumors than did non-immunized controls, but those immunized with low dosages showed a markedly *increased *tumor incidence. This facilitated growth was abolished by irradiation of the mice with 450 rads 24 hours prior to challenge with active virus.

More studies could be discussed, but I have cited enough to establish that immune efficacy is biphasically related to deliberately induced, *in situ*, autochthonous tumors. However, the biphasic curve is not merely a function of a *tumor *response to varied levels of immunity; normal skin allografts show a very similar phenomenon. Chai noted the phenomenon when creating inbred strains of rabbit [33]. During routine skin grafting he found that if two animals were genetically very similar, albeit not identical, they might accept reciprocal skin grafts but nonetheless mount a chronic inflammatory reaction that resulted in the grafts developing a long lasting, chronic hyperplasia. Thus, in rabbit skingrafts as in tumors, a mild immune-reaction stimulated growth, but a larger reaction was destructive.

The next question is, do all or most sporadic tumors have sufficient immunogenicity to produce a similar biphasic response curve?

### A biphasic effect of immunity relative to spontaneous tumors?

Spontaneously arising rodent tumors, *i.e*. tumors that arise without a known cause, seem at first glance to be non-immunogenic as judged by the classical test for their growth as implants in immunized, syngeneic hosts. Certainly the growth of challenge implants of these tumors is not inhibited in putatively immunized hosts. However, in the paper most often cited as demonstrating the non-immunogenicity of spontaneous tumors [34], the authors noted that, in seven out of seven cases, each using a different spontaneous tumor, the challenge tumors in the putatively immunized mice grew *better *than did the controls. Since this work was done before the immunostimulation theory had gained any traction, the authors dismissed the result as some type of artifact. I believe it suggests that even spontaneous tumors in the mouse usually cause, when transplanted to immunologically competent animals, at least some small degree of immune reaction – not a tumor-inhibitory reaction, but at least the tumor is noticed by the immunological mechanism. An increased incidence of various spontaneous tumors in immunodepressed animals also suggests that spontaneous tumors usually have some immunogenicity [35].

The above observations, as well as the results already cited in connection with the more immunogenic, deliberately-induced tumors, suggest that a biphasic effect may be expected. However, the difficulty of working with sporadic tumors renders conclusions weak and rather tenuous.

If one is willing to call Kaposi's sarcoma a spontaneous tumor, its incidence may be instructive. This tumor is a common feature of the acquired immunodeficiency syndrome (AIDS), *but it flares as recovery from immunosuppression occurs during effective AIDS treatment *[36]. This suggests that, although it grows best in the immunocompromised patient, Kaposi's sarcoma may not be caused by decreased immunosurveillance, but rather by a reduction in the HIV patient of the normal immune capacity to a more optimal but still positive level for supporting tumor growth. A reasonable interpretation is that this tumor grows best when the immune capacity of the host is less than normal, *but not too low*. As with any clinical observation, to an even greater degree than in mouse work, any interpretation is merely the best bet among many known and unknown possible confounders.

Consider what is probably the best known and one of the earliest examples suggesting immunosurveillance: the high incidence of some tumors, particularly skin tumors, in patients with immunodeficiency induced to facilitate kidney transplantation [37]. Assuming that immunosuppression is the proximal cause of the phenomenon, lack of surveillance is the logical explanation for the data unless one has in mind the probable biphasic nature of the immune effect. However, if the effect of the immune reaction on primary tumors is biphasic and if the optimum level of host immune-capacity (for tumor growth) varies not only from tumor to tumor but from one tumor *type *to another, one need not invoke a tumor-inhibiting surveillance. Let us assume, in the case of kidney transplant patients, that the excess of skin tumors did indeed result from the reduction of the normal immune capacity. The elevated tumor incidence could be easily interpreted as being caused, not by reduced surveillance, but by positive stimulation by the residual immune reaction, now reduced in such patients to a more nearly optimal level for positive tumor-facilitation. This interpretation assumes that had the immune capacities of the patients been still further reduced, perhaps to nil, the incidence of skin tumors would have again declined. Skin tumors may be particularly facilitated by immunodeficiency because the skin has an unusually active immune mechanism; such tumors would, according to the facilitation hypothesis, grow better if the unusually high immune reactivity were reduced. Other tumor types, such as mammary cancers and rectal carcinomas, usually find the reduced immune capacity in the kidney-transplant patient to be even further from their immunological needs than is the normal immune capacity, probably because of a postulated lesser innate tumor-immunogenicity and/or their arisal in a less immunologically active site. Hence the *lower *than expected incidence of these tumors in immunocrippled patients [38,39].

### Oncogenesis in scid and nude mice and tests for surveillance

Many studies have been interpreted to support the immunosurveillance idea. For example, Engel *et al*. [40] showed that MCA-induced sarcomas that arose in immunocrippled scid-mice grew poorly when transplanted to normal syngeneic hosts as compared to tumors that had been induced in immunocompetent hosts. They argued, quite logically, that immunoselection had eliminated highly immunogenic cells in the competent primary hosts (surveillance) while such cells were allowed to persist in tumors that arose in the crippled mice. However, these data can be reinterpreted, as follows, to be compatible with the data supporting the immunostimulation hypothesis.

Remember that in the Prehn/Bartlett experiment there was apparent selection toward a positive, optimal level of immunogenicity (for tumor growth) [19]. In the Engel experiment [40], selection in the immunocompetent primary hosts would presumably also have been toward an optimal level, *i.e*. toward the level of immunogenicity best for tumor growth in mice with that host's immune capacity. Therefore, according to the facilitation interpretation, the tumor cells from the competent hosts had been selected for more optimal immunogenicity for tumor growth in normal immunocompetent mice; selection in the immunocrippled donors, on the other hand, was for cells that would grow best when the immune reactivity was lower. Thus, the tumor cells obtained from the immunocompetent hosts exhibited better growth when cells of each type were transplanted into immunocompetent recipients. According to the facilitation hypothesis, the selections were not dependent upon inhibiting or killing the less well-adapted cells, but rather upon facilitating the better adapted. This is, I think, more than a mere semantic difference.

This interpretation of the Engel data [40] seems preferable to the surveillance interpretation, not only because it meshes with the data supporting the immunostimulation hypothesis, but also because it offers an explanation for an otherwise inexplicable observation that the authors themselves noted; namely, that the tumors induced in the crippled mice grew more slowly, when transplanted to secondary crippled recipients, than did the tumors obtained from the competent primary hosts [40]. The surveillance interpretation offers no explanation for this; but according to the immunostimulation interpretation, tumors that originated in the immunocrippled scid mice, because of the weakness of the immune response, would have been subjected to little or no immunoselection for faster growth and progression. In contrast, tumors that had originated in immunocompetent hosts would have undergone a selection for progression and increased malignancy and so were better able to thrive when transplanted into the immunocrippled secondary hosts. (The probable effect of immunity in promoting progression will be discussed shortly).

A similar argument in favor of the facilitation hypothesis can be made even if the immune crippling is quite severe. Svane *et al*. [41] compared oncogenesis in nude mice with that in normal immunocompetent mice. I suggest that the excess susceptibility of the nudes could have been caused by their low but still positive immune capacity being somewhat nearer the optimum level for growth of these highly immunogenic tumors, rather than by a lack of surveillance. Notwithstanding the general acceptance of xenografts and the lack of detectable immunological memory, highly immunogenic, and only highly immunogenic, tumor-implants grew significantly better in irradiated than in non-irradiated nude mice, suggesting that these mice retain a low but positive primary immune capacity [42]. Thus, for some very immunogenic tumors, the very low immune capacity of nude mice is apparently greater than is optimal for their growth.

Although the immune capacity of the nudes in the Svane experiment [41] was probably nearer the postulated optimal level for growth of the tumors as compared to the normals, I suggest, because of the great immunogenicity of the resulting tumors, that the immune capacity of the nudes may actually have been less than optimum. Thus, there may have been a selective pressure in favor of those tumors that had a compensatingly greater immunogenicity [42]. These considerations suggest, as is apparently true for Kaposi's sarcoma, that a *partial *restoration of the immune system in the immunodeficient hosts might have increased the tumor incidence.  Alternatively, a lower dosage of carcinogen might have had a similar effect [43].

On the other hand, the immune capacity of nudes was indeed *less *than was optimal for MCA or dibenzanthracine-induced skin carcinogenesis; both Gershwin *et al*. [44], and Outzen [[30], review] reported that papillomas were induced more easily in normals or in immunologically restored nudes than they were in nudes. These findings again suggest that the optimum immune capacity for tumor support varies from one tumor type to another; thus, human skin tumors apparently thrive at the lowered levels of immune capacity found in the kidney-transplant patient, while human mammary and rectal carcinomas seem to grow relatively poorly if the immune capacity of the patient is lowered [38,39].

It seems probable that any data that seem to show immunosurveillance of primary *in situ *tumors can be reinterpreted, by similar means, to be consistent with the immunostimulation idea. To demonstrate immunosurveillance rigorously by an increased tumor incidence in immunodepressed subjects, one must, I think, also show that there would still be an increased tumor incidence if the immune capacity of the host were further lowered, perhaps all the way to nil. Without knowing where on Fig. 1 the data actually lie, interpretation is difficult.

There seem to be sufficient data to show clearly that highly immunogenic tumors, given proper experimental parameters, can sometimes occur with greater speed and frequency in immunodeficient subjects [37,41]. Recently, data accumulated showing an increase of *spontaneous *tumors in immunodeficient 129 mice lacking RAG2 and or STAT1 [35]. This important result is rather surprising inasmuch as past work has not suggested that spontaneous tumors would appear much more readily in immunodeficient than in normal mice [26]. Perhaps we need to consider whether the higher tumor incidence was entirely caused by the immune deficit. Most spontaneous tumors are supposedly only weakly immunogenic. Thus, I would have expected, according to the facilitation theory, that the optimum host immune capacity for the growth of such tumors might have been higher than probably existed in the immunodeficient 129 mice. Would the incidence of spontaneous tumors have been greater if the immune deficit in the 129 mice been *partially *corrected?

### Does immunity facilitate tumor progression?

That immunity may indeed promote dedifferentiation and progression has been suggested by a number of observations. Progression is commonly observed when tumors are transplanted serially in immunocompetent animals, but seems to be much delayed or lacking in immunodeprived hosts [45-47]. Some tumors may even become *more *differentiated when passaged in athymic nude mice [48]. Hammond passaged small-cell lung carcinomas from inbred hamsters into animals of differing immune capacities [49]. Since his important paper is difficult to obtain, I shall quote his conclusions in full: *"These studies show a previously undescribed immune response-related modulating influence upon classic tumor progression in vivo; the rate and degree of dedifferentiation during tumor progression is directly related to the level of host immunocompetence. Immunodepression favors maintenance of the differentiated state, but normal or elevated immunoreactivity is associated with progressive dedifferentiation." *More recently, de Visser *et al*. have presented evidence for B-cell-dependent tumor progression [50] and Daniel *et al*. have shown that CD4+ T cells can enhance skin cancer progression [51]. Much further work will be necessary to confirm the effect of immunity on tumor progression and to determine whether or not such an effect is, like the effect on tumor growth, biphasic.

### Mechanisms and philosophical considerations

Stutman [26], in a very comprehensive and heroic review of studies on the carcinogenic effects of varying host immune capacities, concluded that there was no net evidence in favor of either immunostimulation or immunosurveillance. At the time of that review, it was probably not realized that the effect of the immune response on tumors is biphasic in such a way that an alteration in the magnitude of the normal immune capacity could change a less-than-optimal level of immunity (for tumor growth) to a more-than-optimal or vice versa. Thus, in Fig. 1, moving the quantity of immune reactants from point *b *to point *d *or from point *a *to point *e *would have little effect on tumor growth. Since both lower-than-optimal and higher-than-optimal immune capacity levels might result in much the same tumor incidence, little or no consistent effect on tumor behavior might be seen in consequence of changes in the immune capacities of the primary hosts. The biphasic nature of the immune response in relation to tumor growth would vastly complicate the interpretation of most experiments and could help account for the lack of overall effect noted by Stutman [26].

What could account for the evolution of an immune system that, at moderate or low levels of reaction, apparently promotes the growth of primary *in situ *tumors? It is logical to speculate that the vertebrate immune system was selected, in part, by invading viruses, bacteria and parasites for *their own benefit*, not for the benefit of the host. Therefore the system was probably selected, at least initially, to be helpful and even stimulating to the foreign invaders when these arrived in small numbers, but to inhibit the invaders if and when the invasion became larger and more life-threatening [52]; few infectious invaders would be benefited by rapid death of the host. A primary *in situ *tumor begins as a very small invader and is perhaps seen initially by the immune mechanism much as a tiny parasitical infection might be seen; thus, it may likewise be facilitated to grow, rather than be inhibited, by whatever weak immune reaction may be produced. Indeed, it has been shown that a very tiny tumor inoculum may grow, even in a specifically immunized mouse, when a larger tumor implant would be rejected, a phenomenon known as *sneaking through *[53].

The cellular and molecular bases of the facilitation phenomenon are indubitably complex. I am persuaded by the arguments of Sonnenschein and Soto that proliferation is the cellular default state [54]; therefore, the apparent facilitation of tumor growth and progression by immune reactants must, in actuality, be caused by interference with normal, presumably non-immunological, inhibitors of tissue and tumor growth. There are many data, as discussed by Osgood [55], to the effect that the less differentiated cells in any organ or lesion are regulated by their more differentiated progeny. There followed the concept of the chalone, which actively inhibits the less differentiated cells and is produced by the more differentiated [56]. A relative loss of the more differentiated tumor cells is a critical part of histological tumor-grading. Since expansion of any lesion seems to depend upon a lessening of the inhibiting influence of the more differentiated cells, it is evident that any mechanism that produces less differentiation, or that interferes with signalling from the more differentiated to the less differentiated, would promote tumor-growth and progression. Perhaps, as a speculation, the immune reaction is such a mechanism.

Tumor facilitation can apparently be mediated by any of a large number of immune mediators including antibody [31], T cells and their cytokines [57], macrophages [58] and NK cells [59]. Epidermal growth factor has been shown to stimulate tumor growth at picomolar concentrations but to cause inhibition at nanomolar [60]. There has been much recent interest in the role of inflammation in promoting oncogenesis [61]; and as I have already mentioned, the inflammation associated with a mildly disparate skin-allograft can produce chronic hyperplasia in the graft [33].

## Conclusion

In view of the biphasic curve, it seems that the hypothesis of immunosurveillance, at least as originally conceived, must be discarded. The demonstration of an increased tumor incidence after some degree, even a severe degree, of immunosuppression cannot prove that there might not have been a lower rather than a higher tumor incidence had the immunosuppression been more complete. The reality of the biphasic curve suggests the possibility, even probability, that some level of immune reaction may be necessary for tumor growth in vivo. At least this hypothesis cannot, I believe, be excluded by any presently available data.

Even if a facilitation phenomenon might initially be necessary for the growth of in situ tumors, the immune reaction might develop sufficient strength during later phases of tumor growth to become inhibitory. In this sense, the two hypotheses, facilitation and surveillance, are not necessarily mutually exclusive if temporally displaced. Could actual toxicity to a tumor sometimes follow initial tumor-facilitation? Probably not; the fact that even highly immunogenic, MCA-induced mouse sarcomas are facilitated in situ by the level of immunity they induce [19] suggests that the eventual development of a higher tumor-inhibitory level of immunity is, in the case of most tumors, very unlikely.

For a rather different view of the role of immunity in cancer, see the review by Robert D. Schreiber [35].

## Competing interests

The author(s) declare that they have no competing interests.
